# NT-proBNP and BNP as Biomarkers for Preeclampsia: A Systematic Review and Meta-Analysis

**DOI:** 10.3390/ijms26136272

**Published:** 2025-06-28

**Authors:** Viorela-Elena Suciu, Daniel-Corneliu Leucuța, Andrei Mihai Măluțan, Cristian Iuhas, Mihaela Oancea, Carmen Elena Bucuri, Maria Patricia Roman, Cristina Ormindean, Dan Mihu, Răzvan Ciortea

**Affiliations:** 1 2nd Department of Obstetrics and Gynecology, “Iuliu Hațieganu” University of Medicine and Pharmacy, 400610 Cluj-Napoca, Romania; suciu.viorela.elena@elearn.umfcluj.ro (V.-E.S.); amalutan@umfcluj.ro (A.M.M.); cristian.iuhas@umfcluj.ro (C.I.); mihaela.oancea@umfcluj.ro (M.O.); bucuri.carmen@umfcluj.ro (C.E.B.); rada.maria@umfcluj.ro (M.P.R.); ormindean.cristina@elearn.umfcluj.ro (C.O.); dan.mihu@umfcluj.ro (D.M.); ciortea.razvan@umfcluj.ro (R.C.); 2 Department of Medical Informatics and Biostatistics, “Iuliu Hațieganu” University of Medicine and Pharmacy, 400349 Cluj-Napoca, Romania; 3 Emergency Military Clinical Hospital “Dr. Constantin Papilian”, 400610 Cluj-Napoca, Romania

**Keywords:** preeclampsia, biomarkers, brain natriuretic peptide

## Abstract

The aim of this study was to evaluate and synthesize the existing evidence on N-terminal pro-brain natriuretic peptide (NT-proBNP) and brain natriuretic peptide (BNP) as biomarkers for preeclampsia as compared with a healthy pregnant group, but also comparing them in early-onset preeclampsia (EOP) versus late-onset preeclampsia (LOP). Five electronic databases, PubMed, EMBASE, Web of Science, Scopus, and LILACS, were searched for studies on pregnant women comparing NT-proBNP and BNP levels in preeclampsia vs. healthy pregnancies and EOP vs. LOP. From the 752 identified records, 31 studies were included in the review, referring to 3915 participants. When comparing PE to healthy pregnancies and EOP to LOP, there was a considerable increase in NT-proBNP levels in the PE group, respectively, in EOP: 206.19 pg/mL (95% CI 139.68–272.69) (*p* ≤ 0.001) in the PE group, and 182.42 pg/mL (95% CI 99.65–265.19) (*p* ≤ 0.001) in the EOP group. Regarding BNP, the levels were higher in the PE group (30.13 (95% CI 17.22–43.04), *p* ≤ 0.001), respectively in the EOP group (33.35 pg/mL (95% CI 20.26–46.43), *p* ≤ 0.001). NT-proBNP and BNP levels are consistently elevated in preeclampsia compared to healthy pregnancies and in EOP compared to LOP.

## 1. Introduction

Preeclampsia (PE) represents a pregnancy-specific disease with a prevalence of 2–6% and is strongly correlated with significant morbidity and mortality [1]. It is characterized by new-onset hypertension after 20 weeks of gestation, proteinuria, and/or organ dysfunction, the definitive treatment being the delivery with complete resolution of the symptoms by 12 weeks after delivery [2].

This condition may be life-threatening due to the multiorgan system impact. The severe forms of PE may be associated with hepatic, cardiac, renal, neurological, hematological, or placental dysfunction [3]. Moreover, PE is a significant contributor to neonatal morbidity and perinatal mortality. The fetuses from mothers with PE may present intrauterine growth restriction, stillbirth, and preterm delivery associated with long-term complications, such as cerebral palsy or chronic pulmonary hypertension. Furthermore, the unfavorable intrauterine conditions in PE are believed to play a role in several conditions that could appear during childhood and adulthood, such as hypertension or other cardiovascular diseases, obesity, delayed physical development, or brain anatomical alterations similar to those of autistic children [4,5,6]. Therefore, this condition becomes not only a medical challenge but also a financial challenge for the healthcare system.

The main theory accepted in the development of preeclampsia is represented by uteroplacental ischemia, which can determine placental infarctions followed by toxins released into the maternal system. It has been demonstrated that the ischemia is caused by a defect in placentation but also by an abnormal transformation of the spiral arteries [3,7]. Overall, the pathophysiology of PE is not fully understood, but it is recognized that it represents a disorder of trophoblastic differentiation and invasion of the maternal spiral arteries, probably associated with other mediators or mechanisms, such as pro- and anti-angiogenic biomarkers, inflammatory mediators, genetic predisposition, or autoimmune involvement [7,8].

In recent years, many studies have focused on personalized medicine, trying to identify molecular mechanisms and biomarkers in order to predict the onset of PE or the severity and consequences of this condition. Moreover, biomarkers offer valuable insights into their pathophysiology, enabling early identification of at-risk individuals and facilitating targeted interventions. Two of the most promising options are the markers of cardiovascular stress: N-terminal pro-brain natriuretic peptide (NT-proBNP) and brain natriuretic peptide (BNP).

In a normal pregnancy, cardiac output and maternal heart rate increase, but vascular resistance and blood pressure decrease. In patients with PE, these physiological adaptations do not occur completely, resulting in cardiac dysfunction, which causes the secretion of some markers by cardiomyocytes, especially type B natriuretic peptide (BNP, NT-proBNP) [9].

BNP (32 amino acids) is a peptide synthesized by cardiac myocytes in response to increased intracardiac pressure and cardiac chamber enlargement, deriving from its precursor—proBNP (108 amino acids). BNP is an active molecule and induces vasodilation, natriuresis, and diuresis, in contrast to the NT-proBNP (76 amino acids), which is inactive [10,11]. Studies have shown that NT-proBNP is a more sensitive marker for early cardiac dysfunction than BNP [12]. Currently, NT-proBNP is used in the general population as a biomarker for heart failure, playing a role in diagnosis and prognosis [13]. However, recent studies have shown that NT-proBNP levels are elevated in pregnant women with PE, with even higher levels in early-onset PE (EOP) (<34 weeks of gestation) compared to late-onset PE (LOP) (≥34 weeks of gestation) [14]. Moreover, the levels appear higher in the umbilical vein at birth in infants delivered by mothers with preeclampsia [15].

To our knowledge, there is no published systematic review to evaluate NT-proBNP and BNP in relation to preeclampsia. Thus, this study aimed to assess and synthesize the existing evidence on NT-proBNP and BNP as biomarkers for preeclampsia compared with a healthy pregnant group but also compare them in EOP versus LOP.

## 2. Methods

This systematic review and meta-analysis were reported in accordance with the Preferred Reporting Items for Systematic Reviews and Meta-Analyses (PRISMA) guidelines.

A comprehensive literature search was conducted across five electronic databases: PubMed, EMBASE, Web of Science, Scopus, and LILACS. The search strategy was based on the PICO framework: Population (pregnant women), Intervention/Comparator (preeclampsia vs. healthy pregnancies or EOP vs. LOP), and Outcomes (NT-proBNP and BNP levels). The complete search strategy is presented in the Supplementary File in Supplementary Table S1. No restrictions were placed on language or publication year. Two independent reviewers screened the titles and abstracts of retrieved articles (V.E.S., D.C.L.). The inclusion criteria comprised pregnant women with a singleton pregnancy diagnosed with PE. Exclusion criteria included twin or multiple pregnancies and women with chronic hypertension or gestational hypertension. Studies meeting the inclusion criteria were further evaluated in full-text form (V.E.S., D.C.L.). Disagreements between reviewers were resolved through discussion until a consensus was reached (V.E.S., D.C.L. and A.M.M.).

Data were independently extracted by the authors using a standardized form (V.E.S., D.C.L. and A.M.M.). Extracted information included the following variables: Study characteristics: country, region, study design, study period, number of cases and controls; Participant characteristics: number of participants, age, parity, body mass index (BMI), gestational week at diagnosis, systolic blood pressure (SBP), diastolic blood pressure (DBP), gestational age at delivery, birth weight of the newborn, definitions of preeclampsia, values of NT pro-BNP and BNP, comparison analyzed in the study (PE vs. healthy, EOP vs. LOP).

In cases of discrepancies concerning extracted data, the information was rechecked against the original articles to ensure accuracy. When numerical values (e.g., means, standard deviations) were not directly reported, they were calculated from medians, quartiles, ranges, or standard errors using validated methods. Values presented in graphical formats were extracted using WebPlotDigitizer, version 4.8. For subgroups with reported means and standard deviations (SDs), these values were pooled to estimate the overall group mean and SD when needed.

The methodological quality of the included studies was evaluated using the Newcastle-Ottawa Scale (NOS) by two independent reviewers. Discrepancies in the quality assessment were resolved by discussion. Studies were rated based on selection, comparability, and outcome domains.

Meta-analyses were conducted using the mean difference as the effect size. Random-effect models were used, with the restricted maximum likelihood (REML) method for parameter estimation. Heterogeneity was quantified using the Cochrane guidelines, specifically the I^2^ statistic, the Q-test, and its corresponding *p*-value.

Publication bias was assessed using funnel plots and the Egger test. Sensitivity analyses were conducted using the leave-one-out method to evaluate the robustness of the results.

All analyses were performed using statistical software, and the findings were reported in accordance with PRISMA guidelines.

## 3. Results

**Figure 1:**
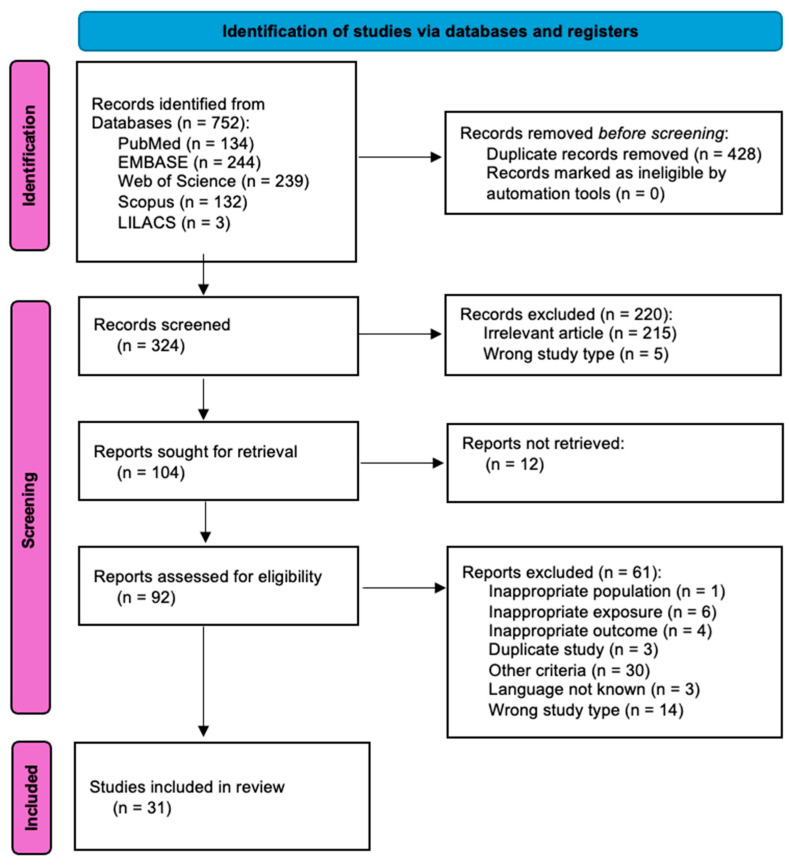
PRISMA flowchart reporting identification, screening, selection, and inclusion of studies in the systematic review.

The PRISMA diagram outlines the process of study selection for a systematic review in the medical field (Figure 1). Initially, 752 records were identified from five databases, including PubMed, EMBASE, Web of Science, Scopus, and LILACS. Before screening, 428 duplicate records were removed, leaving 324 records for screening. Of these, 220 were excluded due to irrelevance (215 articles) or the wrong study type (5). The remaining 104 reports were sought for retrieval, but 12 could not be retrieved. Of the 92 reports assessed for eligibility, 61 were excluded for reasons such as inappropriate population (1), exposure (6), or outcomes (4), as well as duplicate studies (3), other criteria (30), language issues (3), and wrong study types (14). Ultimately, 31 studies were included in the final review, referring to 3915 participants [14,16,17,18,19,20,21,22,23,24,25,26,27,28,29,30,31,32,33,34,35,36,37,38,39,40,41,42,43,44,45].

The study characteristics are presented in Supplementary Table S2.

The included studies comprised investigations from diverse continents, including Europe, Asia, South America, and the USA. The study designs varied, encompassing cross-sectional, case-control, prospective cohort, and retrospective approaches. Participants included pregnant women with a wide age range, from 20 to 40 years. Gestational weeks at diagnosis ranged from 20 to 39 weeks, and gestational weeks at delivery ranged from 24 to 40 weeks. The diagnostic criteria for PE were based on international guidelines definitions, including the ones from the American College of Obstetricians and Gynecologists (ACOG), the International Society for the Study of Hypertension in Pregnancy (ISSHP), or the National High Blood Pressure Education Program’s Working Group on High Blood Pressure in Pregnancy (NHBPEP). Some studies did not indicate the guidelines but presented criteria for PE that are similar to the current guidelines. BNP and NT-proBNP levels were measured using diverse techniques such as radioimmunoassay, fluorescence immunoassay, or Enzyme-Linked Immunosorbent Assay (ELISA). Most investigators sampled the biomarkers at the time of PE diagnosis.

This diversity in study design, population characteristics, and measurement strategies reflects the broad range of methodologies employed to investigate the association between BNP, NT-proBNP levels, and PE.

### 3.1. NT-proBNP

NT-proBNP was assessed in two comparisons: preeclampsia versus healthy patients—as observed in 14 studies, and EOP versus LOP—as observed in 10 studies.

#### 3.1.1. Preeclampsia vs. Healthy

**Figure 2:**
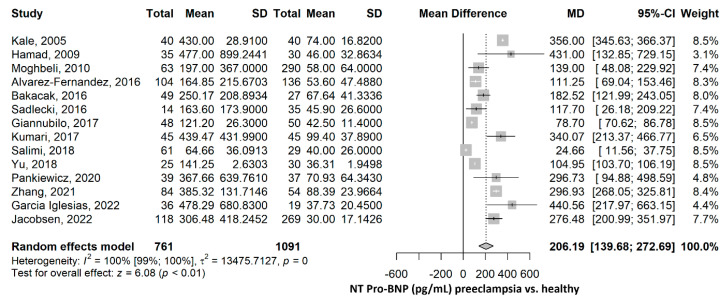
NT-proBNP mean difference meta-analysis between preeclampsia and healthy patients. NT, N terminal; BNP, brain natriuretic peptide; SD, standard deviation; MD, mean difference; CI, confidence interval [28,29,30,31,32,33,34,35,36,39,40,42,43,44].

The pooled NT-proBNP mean was 206.19 pg/mL (95% CI 139.68–272.69), *p* ≤ 0.001, higher in the PE group compared to the healthy group (Figure 2). The heterogeneity was considerable, with an I^2^ of 99.5% (95% CI 99.4–99.6%), *p* ≤ 0.001. Nevertheless, all studies were statistically significant, and all showed higher NT-proBNP values in the PE group compared to the healthy group. Furthermore, a leave-one-out sensitivity analysis was performed, where no matter which study was excluded, the final result remained statistically significant, with higher NT-proBNP values in the PE group compared to the healthy group, thus sustaining the robustness of our results (Supplementary Figure S1). After the exclusion of each of the studies, the heterogeneity remained high, ranging from 97% to 100%.

#### 3.1.2. EOP vs. LOP

**Figure 3:**
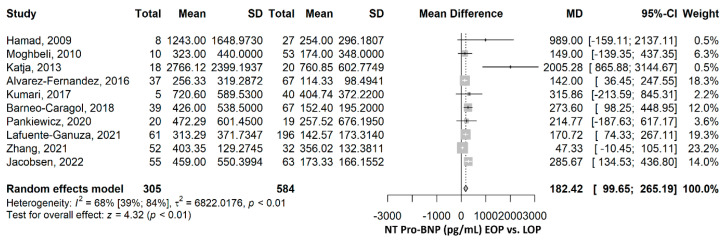
NT-proBNP comparison between early-onset preeclampsia (EOP) and late-onset preeclampsia (LOP). NT, N terminal; BNP, brain natriuretic peptide; SD, standard deviation; MD, mean difference; CI, confidence interval [14,28,31,34,35,36,38,41,42,44].

The mean NT-proBNP was 182.42 pg/mL (95% CI 99.65–265.19), *p* ≤ 0.001, higher in the EOP group compared to the LOP group (Figure 3). The heterogeneity was substantial, and we found an I^2^ of 68.3% (95% CI 38.6–83.6%), and the Q test for heterogeneity gave *p* ≤ 0.001. The mean differences between all studies were higher in the EOP group than in the LOP group. The sensitivity analyses by the leave-one-out method offered significantly higher mean NT-proBNP in the EOP compared to the LOP group, sustaining the robustness of the results (Supplementary Figure S2). After the exclusion of each of the studies, the heterogeneity ranged from 68% to 72%, for most of the studies, except for Katja, 2013 [38], and Zhang, 2021 [44], with I^2^ of 55% and 47%.

### 3.2. BNP

BNP was assessed in two comparisons: preeclampsia versus healthy patients—as observed in 11 studies, and EOP versus LOP—as observed in two studies.

#### 3.2.1. Preeclampsia vs. Healthy

**Figure 4:**
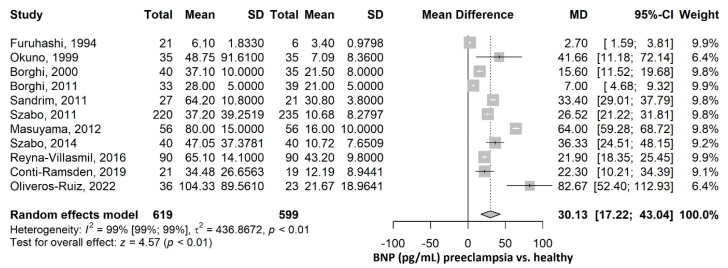
BNP mean difference meta-analysis between preeclampsia and healthy patients. BNP, brain natriuretic peptide; SD, standard deviation; MD, mean difference; CI, confidence interval [16,17,18,19,20,21,22,23,24,26,45].

The mean value of BNP (pg/mL) was 30.13 (95% CI 17.22–43.04), *p* ≤ 0.001, higher in the preeclampsia group compared to the healthy group (Figure 4). The heterogeneity was considerable, with an I^2^ of 98.9% (95% CI 98.6–99.1%), *p* ≤ 0.001. Nevertheless, all studies were statistically significant, and all showed higher BNP values in the PE group compared to the healthy group. Furthermore, a leave-one-out sensitivity analysis was performed, where no matter which study was excluded, the final result remained statistically significant, with higher BNP values in the PE group compared to the healthy group, thus sustaining the robustness of our results (Supplementary Figure S3). After the exclusion of each of the studies, the heterogeneity remained high, ranging from 98% to 99%.

#### 3.2.2. EOP vs. LOP

**Figure 5:**
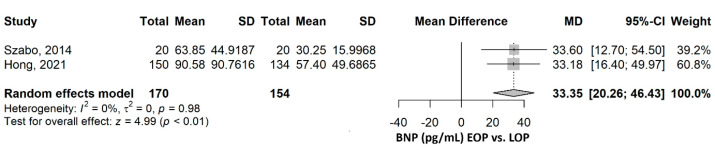
BNP comparison between early-onset preeclampsia (EOP) and late-onset preeclampsia (LOP). BNP, brain natriuretic peptide; SD, standard deviation; MD, mean difference; CI, confidence interval [20,25].

The mean BNP was 33.35 pg/mL (95% CI 20.26–46.43), *p* ≤ 0.001, higher in the EOP compared to the LOP group (Figure 5). The heterogeneity was not important, with an I^2^ of 0%, *p* = 0.976. Excluding any of the two studies in the sensitivity analysis did not influence the direction and significance of the result (Supplementary Figure S4).

### 3.3. Risk of Bias Assessment

Most of the domains of the Newcastle Ottawa Scale were without risk of bias (Supplementary Table S3, Supplementary Figure S5). The case definition and definition accuracy were appropriate since the majority of the studies indicated which guidelines were used to define the presence of PE. Some studies did not indicate the guideline but presented criteria for PE that are similar to current guidelines; thus, the definition can be considered as appropriate. The representativeness of the cases suffered in most of the studies since they did not mention whether the sample was consecutive or representative. This does not necessarily imply they were not representative, since this might have been forgotten to be clarified in Section 2. The comparability is at risk of bias for 68% of the studies (21), while 23% of the studies used matching (usually for two important variables: age and gestational age, and sometimes for body mass index, parity, and smoking habits). The ascertainment of exposure and the use of the same method of ascertainment in both cases and controls were at low risk of bias for all the studies since the standard methods of measuring NT-proBNP or BNP were used for all the participants.

### 3.4. Publication Bias

The Egger test was not significant when comparing preeclampsia vs. healthy patients regarding NT-proBNP (*p* = 0.282; Supplementary Figure S6), while it was significant for BNP (*p* = 0.017; Supplementary Figure S7). When comparing EOP with LOP patients regarding NT-pro-BNP, *p* was 0.006 (Supplementary Figure S8), while BNP could not be computed since the number of studies was low (Supplementary Figure S9).

## 4. Discussion

This meta-analysis and systematic review thoroughly evaluated the levels of BNP and NT-proBNP in preeclampsia in comparison to healthy pregnancies, as well as in early-onset preeclampsia in comparison to late-onset preeclampsia. When comparing PE to healthy pregnancies and EOP to LOP, there was a considerable increase in NT-proBNP and BNP levels. The results were robust and supported by leave-one-out sensitivity analyses, and they were similar across all of the studies that were part of the research. The trustworthiness of the findings was shown by the fact that, despite significant heterogeneity in several comparisons, their direction and significance remained constant.

The high levels of NT-proBNP and BNP in PE compared to healthy pregnancies highlight their potential use in screening and monitoring this disease. The significantly different levels of the studied biomarkers in our study may reflect underlying pathophysiological differences. Thus, these biomarkers might complement clinical classification and potentially improve risk stratification, early diagnosis, and individualized management in the future.

Substantial heterogeneity was observed in this meta-analysis, particularly in NT-proBNP comparisons between preeclampsia and healthy pregnancies (I^2^ > 99%), likely due to differences in study designs, study populations (e.g., maternal age, ethnicity, body mass index, time of sample collection), and biomarker measurement methods. Heterogeneity was lower in EOP versus LOP comparisons, indicating more consistent differences between these subtypes.

Concerning the publication bias apparently observed, there are some possible explanations. As a measure of asymmetry, the Egger test does not fully differentiate publication bias from other asymmetry-causing factors, such as actual population heterogeneity. For instance, the Egger test may mistakenly interpret BNP levels as publication bias if they really differ across several subgroups (such as early vs. late-onset PE). Asymmetry in the published literature is likely caused by a mix of small-study effects, selective reporting (publication bias), and genuine heterogeneity, all of which are likely to be reflected in the significant Egger test result observed in our meta-analysis.

The studies included in this meta-analysis assessed the NT-proBNP or BNP levels at the time of the PE diagnosis. This cross-sectional approach precludes causal inferences for the argument of temporal precedence. Nevertheless, it allows for arguing for the strength of association criteria of causality. We found several studies that looked for a longitudinal assessment of NT-proBNP or BNP levels prior to the development of PE. The literature of this type is limited and heterogeneous in its measurement timing of NT/BNP levels as well as of the outcome measured, or of the selection criteria of the cohort, or of the statistical method used for the assessment of the relationship with the outcome. Therefore, a formal meta-analysis was not feasible.

Hauspurg et al. [46] in a multicenter prospective observational study on 4103 nulliparous women, found that elevated NT-proBNP levels in early pregnancy (first trimester blood sample) were linked to a notably reduced risk of developing hypertensive disorders during pregnancy (including PE), as well as a lower likelihood of new-onset hypertension within 2–7 years postpartum.

Phil et al. [47], in a nested case-control study on 717 nulliparous women, with NT-proBNP measured during the first trimester, found a decreased NT-proBNP in cases with term PE (from 37 weeks + 0 days), but no significant association was revealed with preterm PE (before 37 weeks + 0 days).

Vieira et al. [48], in an international multicenter cohort study on 3940 nulliparous women with singleton pregnancy, with BNP measured between 14 and 16 weeks of gestation (second trimester), in the multivariate logistic regression, did not find a significant association with PE.

Uyar et al. [49], in a prospective study on 68 patients (with diastolic notch and abnormal pulsatility index (PI) compared to ones without diastolic notch and with normal PI), measured NT-proBNP levels within 21–24 weeks (second trimester), and they did not find a significant association with PE.

Junus et al. [50], in a case-control study from a prospectively collected database on about half of the population of pregnant women attending Uppsala University, on 59 participants, with NT-proBNP measured during the second trimester, did not find a significant association with PE.

Bacmeister et al. [51], in a population-based cohort study of 1476 participants with NTproBNP measured during the third trimester, found that elevated levels were associated with a higher risk of developing preeclampsia within 4 weeks but with a decreased risk when the timeframe extended beyond 4 weeks.

The relationship between NT-proBNP/BNP and PE seems to be dynamic in time. Studies measuring NT-proBNP/BNP at the diagnostic time of PE, as found by our meta-analysis, show a direct relationship with PE. The two studies measuring NT-proBNP during the first trimester found an inverse relationship; three studies measuring NT-proBNP during the second trimester did not find a significant relationship; one study, measuring NT-proBNP during the third trimester found elevated levels were associated with a higher risk of developing preeclampsia within 4 weeks but with a decreased risk when the timeframe extended beyond 4 weeks.

A recent study by Sabria et al. [52] suggested that NT-proBNP may contribute to short-term risk stratification in preeclampsia. For example, when combined with the soluble fms-like tyrosine kinase-1 (sFlt-1)/placental growth factor (PlGF) ratio, NT-proBNP has been shown to improve the prediction of the need for delivery within one week in women with suspected PE and elevated angiogenic markers. This finding supports the potential clinical utility of NT-proBNP beyond diagnosis, particularly in informing management decisions and timing of delivery.

Our review found that NT-proBNP and BNP levels are higher in women with preeclampsia and in the early-onset type compared to the late-onset type. Mechanistically, this finding can be explained by increased myocardial wall stress due to the elevated systemic vascular resistance and endothelial dysfunction. These hemodynamic changes induce subclinical cardiac strain and impaired ventricular relaxation. This, in turn, stimulates the release of natriuretic peptides. Their use could support risk stratification at the time of diagnosis and help identify women who may benefit from closer surveillance or management of the delivery timing.

### 4.1. Limitations

It is important to recognize and underline the various limitations of this systematic review and meta-analysis. First, the considerable heterogeneity observed in many of the comparisons, particularly in NT-proBNP levels between preeclampsia and healthy pregnancies, limits the ability to generalize these findings. While random-effects models were used to account for heterogeneity, the underlying causes—such as differences in study populations, methodologies, and diagnostic criteria—could not be adequately addressed.

Second, although we calculated missing means and standard deviations using established methods and extracted data from figures with WebPlotDigitizer, these processes introduce the potential for estimation errors. Similarly, presumptions regarding the homogeneity of subgroups would not necessarily hold true when group-level statistics were estimated using subgroup data.

Third, the Egger test showed publication bias for BNP levels, suggesting that the literature may not contain as many smaller studies with nonsignificant findings. Although we addressed this problem with funnel plots and sensitivity analyses, it is impossible to completely rule out its impact on the pooled findings.

Fourth, because most of the included studies were observational, there were inherent risks of bias, confounding, and methodological quality diversity. Despite using the Newcastle-Ottawa Scale to assess study quality, residual confounding may still affect the pooled results.

Fifth, variations in the definitions of preeclampsia, EOP, and LOP across studies may have impacted the comparability of findings. Standardizing the definitions and measuring procedures should be the goal of future research in order to improve the validity of biomarker investigations in hypertensive disorders of pregnancy.

Finally, possible problems in case representativeness and the lack of methods to address comparability can cause other biases in the results.

### 4.2. Strengths

This systematic review and meta-analysis have several key strengths. The risk of neglecting significant research was reduced by the thorough search technique that was conducted across five main databases. Despite heterogeneity, the accuracy of the results was confirmed by a thorough sensitivity analysis, including leave-one-out techniques. Data comprehensiveness was improved using extraction approaches such as estimating pooled values from subgroups, extracting data from figures using WebPlotDigitizer, and calculating means and standard deviations from medians.

### 4.3. Clinical Utility

This meta-analysis highlights the potential of NT-proBNP and BNP as valuable biomarkers for the detection, risk stratification, and management of PE. Their elevated PE levels in comparison to healthy pregnancies suggest that they might help identify high-risk patients sooner and offer more thorough monitoring. Using these biomarkers may improve the diagnosis and the monitoring of this disease and may lead to personalized treatment strategies, reducing the risks and complications associated with PE.

## 5. Conclusions

This systematic review and meta-analysis demonstrate that NT-proBNP and BNP levels are consistently higher in preeclampsia compared to healthy pregnancies and in EOP compared to LOP. While findings were solid, significant heterogeneity underlines the need for standardized procedures and further validation. Future studies should explore their clinical integration in order to improve maternal and fetal outcomes.

## Data Availability

The data presented in this study are available on request from the corresponding author.

## Supplementary Materials

The following supporting information can be downloaded at: https://www.mdpi.com/article/10.3390/ijms26136272/s1.
