# Supplementary material for: NT-proBNP and BNP as Biomarkers for Preeclampsia: A Systematic Review and Meta-Analysis
Source: Int J Mol Sci. 2025 Jun 28;26(13):6272. doi: 10.3390/ijms26136272 (PMC12250389; doi:10.3390/ijms26136272)
Supplement: Supplementary file 1 [file ijms-26-06272-s001.zip › ijms-3647850-supplementary.pdf]

Search strategies

Supplementary Table S1. Search strategies by database.

**PubMed**
(((("N-terminal"[All Fields] AND "pro"[All Fields]) OR "NT-pro"[All Fields] OR "NT pro"[All Fields]) AND ("BNP"[All Fields] OR "B-type natriuretic peptide"[All Fields])) OR "N-terminal pro-brain natriuretic peptide"[All Fields] OR ("pro brain natriuretic peptide 1 76"[Supplementary Concept] OR "pro brain natriuretic peptide 1 76"[All Fields] OR "NT-probnp"[All Fields] OR "NT probnp"[All Fields]) OR "proBNP"[All Fields] OR "BNP"[All Fields] OR "Brain Natriuretic Peptide"[All Fields]) AND ("pre eclampsia"[MeSH Terms] OR "pre eclampsia"[All Fields] OR "preeclampsia"[All Fields])
**Embase**
(('n-terminal' AND ('pro'/exp OR pro) OR 'nt-pro' OR 'nt pro') AND (bnp OR 'b-type natriuretic peptide'/exp OR 'b-type natriuretic peptide') OR 'n-terminal pro-brain natriuretic peptide'/exp OR 'n-terminal pro-brain natriuretic peptide' OR 'nt probnp' OR probnp OR bnp OR 'brain natriuretic peptide'/exp OR 'brain natriuretic peptide') AND ('preeclampsia'/exp OR preeclampsia) AND 'human'/de AND ('article'/it OR 'review'/it)
**SCOPUS**
TITLE-ABS-KEY ((((("N-terminal" AND pro) OR "NT-Pro" OR "NT pro") AND (bnp OR ("B-type natriuretic peptide"))) OR "N-terminal pro-brain natriuretic peptide" OR nt-probnp OR probnp OR bnp OR "Brain Natriuretic Peptide") AND preeclampsia) AND (LIMIT-TO (DOCTYPE , "ar") OR LIMIT-TO (DOCTYPE , "re")) AND (LIMIT-TO (EXACTKEYWORD , "Human"))
**Web of science**
TS=((((("N-terminal" AND Pro) OR "NT-Pro" OR "NT pro") AND (BNP OR ("B-type natriuretic peptide"))) OR "N-terminal pro-brain natriuretic peptide" OR NT-proBNP OR proBNP OR BNP OR "Brain Natriuretic Peptide") AND preeclampsia)
**LILACS**
tw:((((("N-terminal" AND Pro) OR "NT-Pro" OR "NT pro") AND (BNP OR ("B-type natriuretic peptide"))) OR "N-terminal pro-brain natriuretic peptide" OR NT-proBNP OR proBNP OR BNP OR "Brain Natriuretic Peptide") AND preeclampsia)

Supplementary Table S2. Study characteristics

Study	Country	Region	Study design	Period	Moment	Age (years), mean (SD)/median (IQR) [range] Case/Control	Parity	BMI
Álvarez-Fernández, 2016	Spain	Europe	Retrospective full-blinded cohort study	January 2010-March 2014	Triage with suspicion of PE	<34WG: 34 (30-38)/34 (31-37); >=34WG: 35 (32-37)/34(30-37)	-	<34WG:30 (28-36)/29 (26-34); >=34WG: 32 (28-35)/31 (28-35)
Bakacak, 2016	Turkey	Asia	Prospective cohort study	January 2013-July 2014	At the time of PE diagnosis	-/27.26±5.35	-	-/27.86±5.42
Barneo-Caragol, 2018	Spain	Europe	-	October 2012-June 2016	-	34.3±6.1/33.8±4.5	-	-
Borghi, 2011	Italy	Europe	Cross-sectional observation study	-	Detection of BP abnormalities at a periodic control	32.1±5/31.1±4	1.7±0.6/1.6±0.7	-
Borghi, 2000	Italy	Europe	Case/Control	-	Detection of BP abnormalities at a periodic control	31.4±5/31.2±3	1.72±0.8/1.63±7.0	-
Conti-Ramsden, 2019	UK	Europe	Poster	-	-	-	-	-
Furuhashi, 1994	Japan	Asia	Case/Control	-	-	28.3±5.6/ -	-	-
García Iglesias, 2022	Spain	Europe	Prospective cohort study	18 months	At the time of PE diagnosis	34.05 (5.7)/30.5 (5.9)	-	27.67 (5.33)/27.99 (5.84)
Giannubilo, 2017	Italy	Europe	Prospective observational study	January 2014-December 2015	At the time of PE diagnosis	32.2±1.3/31.7±1.8	-	28.3±6.6/25.2±6.7
Hamad, 2009	Sweden	Europe	-	October 2004-November 2007	At the time of PE diagnosis	31(5)/31(4)	-	-
Hong, 2021	China	Asia	Retrospective study	January 2017-July 2019	-	32.01±5.51/32.06±5.87	-	-
Jacobsen, 2022	Norway	Europe	Cross-sectional	-	-	-/33.8 (30.7-36.4)	-	-
Junus, 2014	Sweden	Europe	Case/Control	-	-	-	-	-
Kale, 2005	Turkey	Asia	Casse/Control	April 2003-October 2004	At the time of PE diagnosis	29.6±5.2/28.5±3.7	1.58±1.21/1.44±1.32	-
Katja, 2013	-	-	Poster	-	-	-	-	-
Kumari, 2017	India	Asia	Prospective observational study	May 2015-May 2016	-	25.31±3.30/25.9±4.67	-	-
Lafuente-Ganuza, 2021	Spain	Europe	Retrospective analysis of a prospectively collected data	February 2016-March 2020	-	34 (28-38)/-	-	30 (26-32)/31 (27-33)
Masuyama, 2012	Japan	Asia	Cross sectional study	-	-	30.7±5.3/30.5±4.8	-	24.5±3.1/24.3±2.7
Moghbeli, 2010	USA	North America	Prospective case-controlled study	2005-2007	-	27.5 (6.8)/27.1 (6.7)	-	28.0 (8.0)/28.8 (7.0)
Okuno, 1999	Japan	Asia	Case/Control	September 1993- August 1995	At the time of PE diagnosis	30.3±5.5/29.8±4.3	-	-
Oliveros-Ruiz, 2022	Mexic	South America	Observational cross-sectional and prospective study	November 2018-December 2019	-	27 (23-35)/26 (19-29)	-	31.6 (27.5-36.1)/29.7 (27.7-36.4)
Pankiewicz, 2020	Poland	Europe	Case/Control	August 2015-April 2018	-	32974±4.49/30.895±5.44	-	31.927±6.85/27.893±4.03
Reyna-Villasmil, 2016	Venezuela	South America	Case/Control	January 2014-February 2016	-	21.9±2.4/22.4±2.3	-	30.0±1.2/29.7±1.2
Sadlecki, 2016	Poland	Europe	-	2013-2015	3rd Trimester	29.1±4.6/29.4±5.0	1.9±1.0/1.8±1.0	25.6±2.0/25.2±2.5
Salimi, 2018	Iran	Asia	Descriptive study	-	-	-	-	-
Sandrim, 2011	Brazil	South America	-	-	-	27.7±5.8/24.5±4.2	-	30.9±6.7/27.7±4.0
Seong, 2011	South Korea	Asia	-	March 2006-June 2007	-	-/30.91±4.49	-	-/24.53±3.26
Szabó, 2014	Hungary	Europe	-	January 2008-December 2010	-	30 (27-36)/31 (28-34)	-	24.0 (23.1-25.6)-24.2 (20.6-25.4)
Szabó, 2011	Hungary	Europe	Case/Control	-	-	30 (27-34)/31 (28-34)	-	-
Yu, 2018	China	Asia	Retrospective study	March 2014-July 2016	-	-/29±6	-	24.9±1.8/23.5±2.1

Zhang, 2021	China	Asia	Retrospective cohort study	August 2015-October 2018	-	-/30.7±3.7	-	-/26.3±2.6

BNP, brain natriuretic peptide; PE, preeclampsia; SD, standard deviation; IQR, interquartile range; BMI, body mass index; WG, weeks of gestation; BP, blood pressure; - , not specified, UK, United Kingdom, USA, United States of America.

Study	Gestational week of diagnosis	SBP	DBP	Weeks of delivery	Birth weight	Defitintion PE	Biomarker	Comparison	Case	Control
Álvarez-Fernández, 2016	<34WG: 31+1(27+4-35+6)/31+0(26+1-32+6); >34WG:37+0(36+0-38+5)/37+5(36+4-39+4)	<34WG:149 (134-156)/137 (114-148); >34WG:143 (135-160)/133 (123-146)	<34WG:98 (91-103)/84 (73-95); >=34WG:94 (89-100)/91 (81-96)	<34WG: 33+5 (31+0-34+1)/38+2(37+1-40+3); >=34WG:38+0(37+0-39+2)/39+3(38+2-40+2)	-	ACOG 2002; Zeisler&el al	NT-proBNP	PE vs. healthy	104	136
Bakacak, 2016	-/37.19±1.71	-/110 (130-85)	-/70 (85-55)	-	/2928.15±542.87	ACOG 2013	NT-proBNP	PE vs. healthy	49	27
Barneo-Caragol, 2018	29.9±2.8/38.0±1.8	-	-	31.3±3.2/38.0±1.8	-	ACOG 2013	NT-proBNP	EOP vs. LOP	39	67
Borghi, 2011	30.0±5/30.5±5	141.0±12/108.5±13	91.1±9/61.9±9	-		NHBPEP 2001	BNP	PE vs. healthy	33	39
Borghi, 2000	28.4±6.0/30.9**±4.0**	140.4±14.0/107.1±12.0	89.1±7.0/61.5±8.0	-	-	NHBPEP 1991	BNP	PE vs. healthy	40	35
Conti-Ramsden, 2019	-	-	-	-	-	-	BNP	PE vs. healthy	21	19
Furuhashi, 1994	38.2±2.8	158.5±(SE)9.6/118.0±6.4	91.2±5.9/73.2±4.9	-	-	-	BNP	PE vs. healthy	21	6
García Iglesias, 2022	34.63 (3.17)/33.13 (2.08)	144.84(15.07)/111/58(11.21)	85.04(10.872)/61.87(9.9)	-	-	ACOG	NT-proBNP	PE vs. healthy	36	19
Giannubilo, 2017	31.2±4.2/30.5±3.6	161.5±7.5/112.3±7.8	110.6±18.1/62.7±9.6	35.2±3.7/39.1±2.1	-	ISSHP	NT-proBNP	PE vs. healthy	48	50
Hamad, 2009	35(4)/33(4)	154(2)/117(2)	99(2)/76(2)	36(4)/40(2)	2563 (979)/3293 (411)	ISSHP	NT-proBNP	PE vs. healthy/ EOP vs. LOP	35/8	30/27
Hong, 2021	33.64±3.37/37.65±1.68	165.92±21.23/158.43±17.42	106.27±15.07/101.47±12.16	-		ACOG 2019	BNP	EOP vs. LOP	150	134
Jacobsen, 2022	-/39.0 (38.7-39.3)	-/120 (114-131)	-/75 (69-82)	-/39.0 (38.7-39.3)	-/3474 (3195-3731)	NHLBI working group	NT-proBNP	PE vs. healthy	118	269
Junus, 2014	-	-	-	-	-	#	NT-proBNP	PE vs. healthy	38	36
Kale, 2005	34.0±4.2/35.9±2.9	154.5±5.6/113.2±4.2	98.2±3.4/70.6±4.8	-	-	NHBPEP	NT-proBNP	PE vs. healthy	40	40
Katja, 2013	-	-	-	-	-	-	NT-proBNP	EOP vs. LOP	18	20
Kumari, 2017	-	-	-	-	-	ACOG 2013	NT-proBNP	PE vs. healthy	45	45
Lafuente-Ganuza, 2021	30 (37-32)/-	-	-	32 (30-33)/-	-	ACOG 2013	NT-proBNP	EOP vs. LOP	61	196
Masuyama, 2012	36.4±1.0/37.0±1.3	169±10/107±16	112±8/68±11	36.4±1.0/37.0±1.3	2216±341/2604±214	Japan Society of Obstetrics and Gynecology	BNP	PE vs. healthy	56	56
Moghbeli, 2010	-	-	-	37 (3.1)/39.5 (1.3)	-	ACOG 2002	NT-proBNP	PE vs. healthy	63	290
Okuno, 1999	30.7±6.4±31.1±5.7	158.5±19.1/107.6±9.9	99.3±11.4±65.3±7.1	-	-	ACOG 1990	BNP	PE vs. healthy	35	35
Oliveros-Ruiz, 2022	-	152 (140-160)/100 (90-100)	90 (90-100)/60 (60-70)	36 (30-38)/38 (37-39)	-	-	BNP	PE vs. healthy	36	23
Pankiewicz, 2020	33.718±3.46/36.882±2.11	-	-	35.28±3.48/39.08±1.12	2344.1±900.86/3377.24±448.45	ESC Guidlines 2011	NT-proBNP	PE vs. healthy	39	37
Reyna-Villasmil, 2016	-	150.2±8.3/104.5±8.0	106.2±8.3/74.3±8.0	38.8±1.0/38.9±1.1	2929±344/3645±390	#	BNP	PE vs. healthy	90	90
Sadlecki, 2016	38±2.8/40±1.1	-	-	-	3298±440/3570±430	#	NT-proBNP	PE vs. healthy	14	35
Salimi, 2018	-	-	-	-	-	ACOG 2013	NT-proBNP	PE vs. healthy	61	29
Sandrim, 2011	34.8±4.9/36.2±1.6	140.3±16.2/110.5±8.9	89.2±12.6/70.5±7.6	34.5±4.7/40.1±2.2	2277±1043/3167±827	NHBPEP	BNP	PE vs. healthy	27	21

Seong, 2011	-	-	-	-	-	NHBPEP 2000	NT-proBNP	PE vs. healthy	41	45	
Szabó, 2014	30 (28-32)/36 (35-37)	175 (170-185)/170 (158-180)	110 (104-120)/107 (98-110)	31 (28-33)/37 (36-38)	1200 (1400-1755)/2940 (2640-3090)	ACOG 2013	BNP	EOP vs. LOP	20	20	
Szabó, 2011	-	-	-	34 (31-37)/39 (38-40)	1850 (1235-2695)/3270 (3020-3660)	ACOG 2002	BNP	PE vs. healthy	220	235	
Yu, 2018	32±6/33±4	157±14/115±8	100±11/65±7	-	-	NHBPEP	NT-proBNP	PE vs. healthy	25	30	
Zhang, 2021	-/38.8±0.9	-	-	-	-/3356.6±381.2	ISSHP 2018	NT-proBNP	PE vs. healthy	84	54	

SBP, systolic blood pression; DBP, diastolic blood pression; PE, preeclampsia; WG, weeks of gestation; -, not specified; NT-proBNP, N-terminal pro–B-type natriuretic peptide; BNP, brain natriuretic peptide; EOP, early onset preeclampsia; LOP, late onset preeclampsia; #, The guideline name was not specified, but the definition of PE is adequate; ACOG, American College of Obstetricians and Gynaecologists; NHBPEP, The National High Blood Pressure Education Program's Working Group on High Blood Pressure in Pregnancy; ISSHP, The International Society for the Study of Hypertension in Pregnancy; NHLBI, The National Heart, Lung, and Blood Institute.

Sensitivity analyses

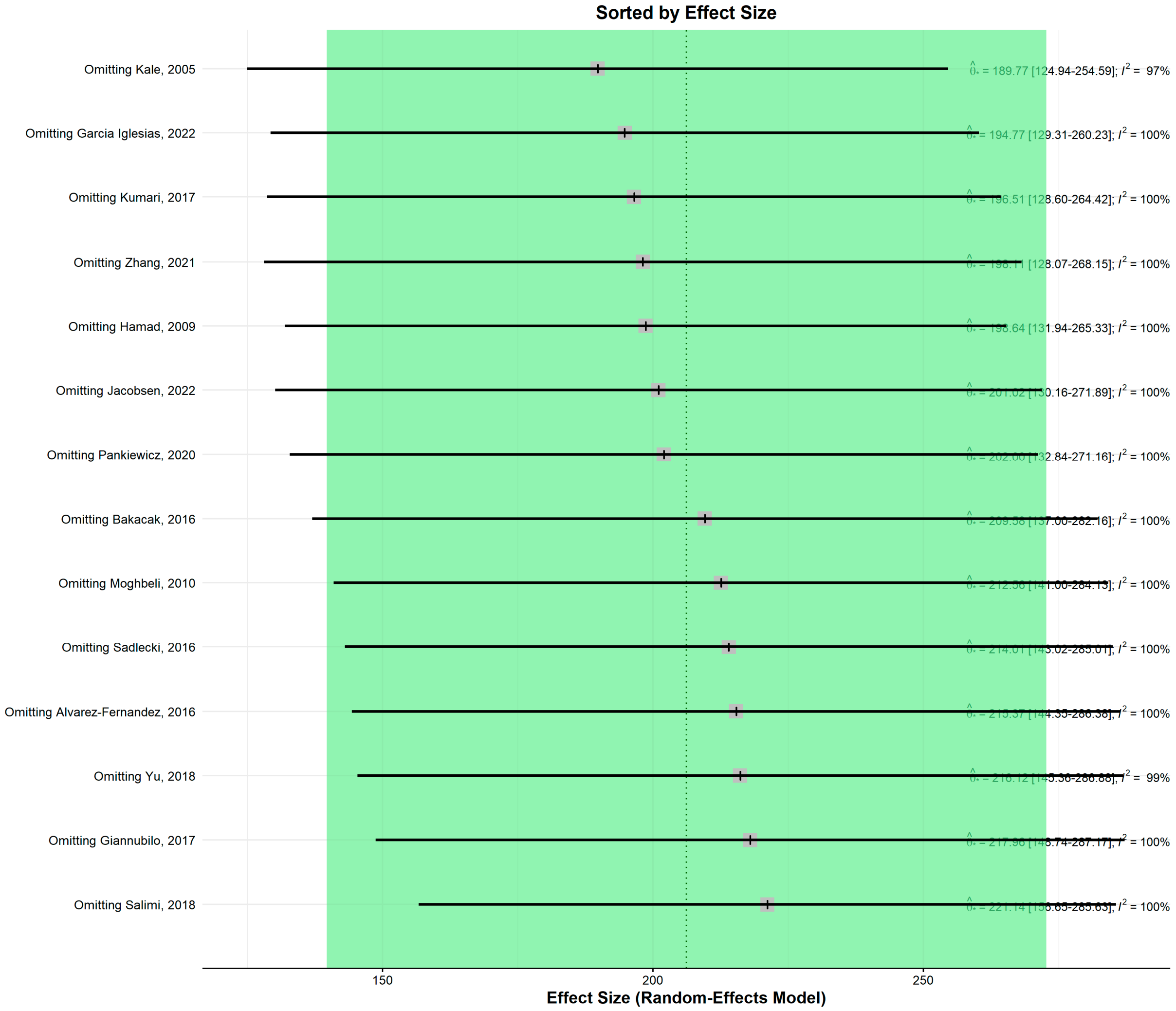

Supplementary Figure S1. Sensitivity analysis for NT Pro-BNP comparison between preeclampsia vs. healthy.

NT, N terminal; BNP, brain natriuretic peptide; Θ, mean difference [95% confidence interval]; I^2, inconsistancy index.

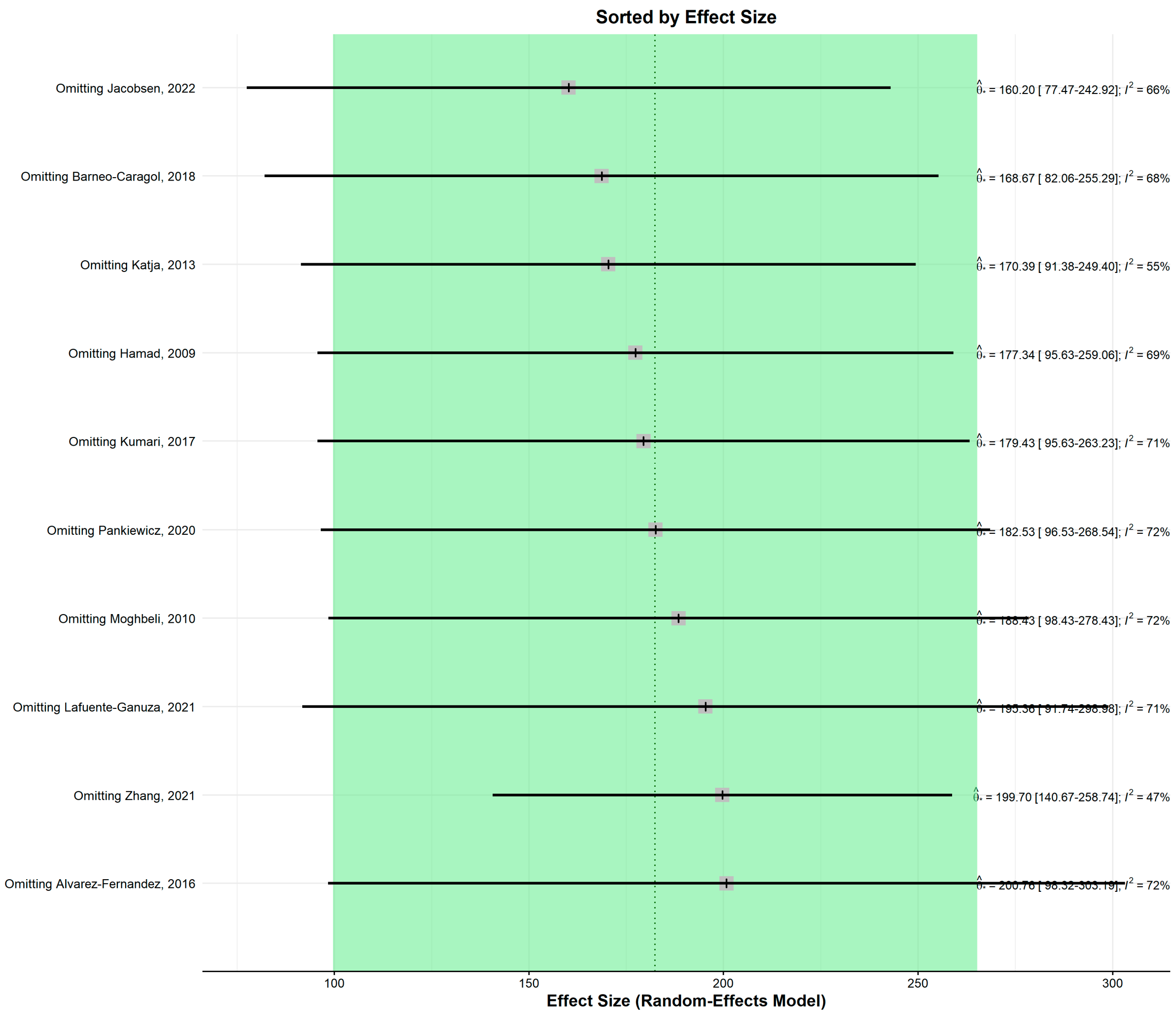

Supplementary Figure S2. Sensitivity analysis for NT Pro-BNP comparison between early onset preeclampsia (EOP) and late onset preeclampsia (LOP).

NT, N terminal; BNP, brain natriuretic peptide; Θ, mean difference [95% confidence interval]; I^2, inconsistancy index.

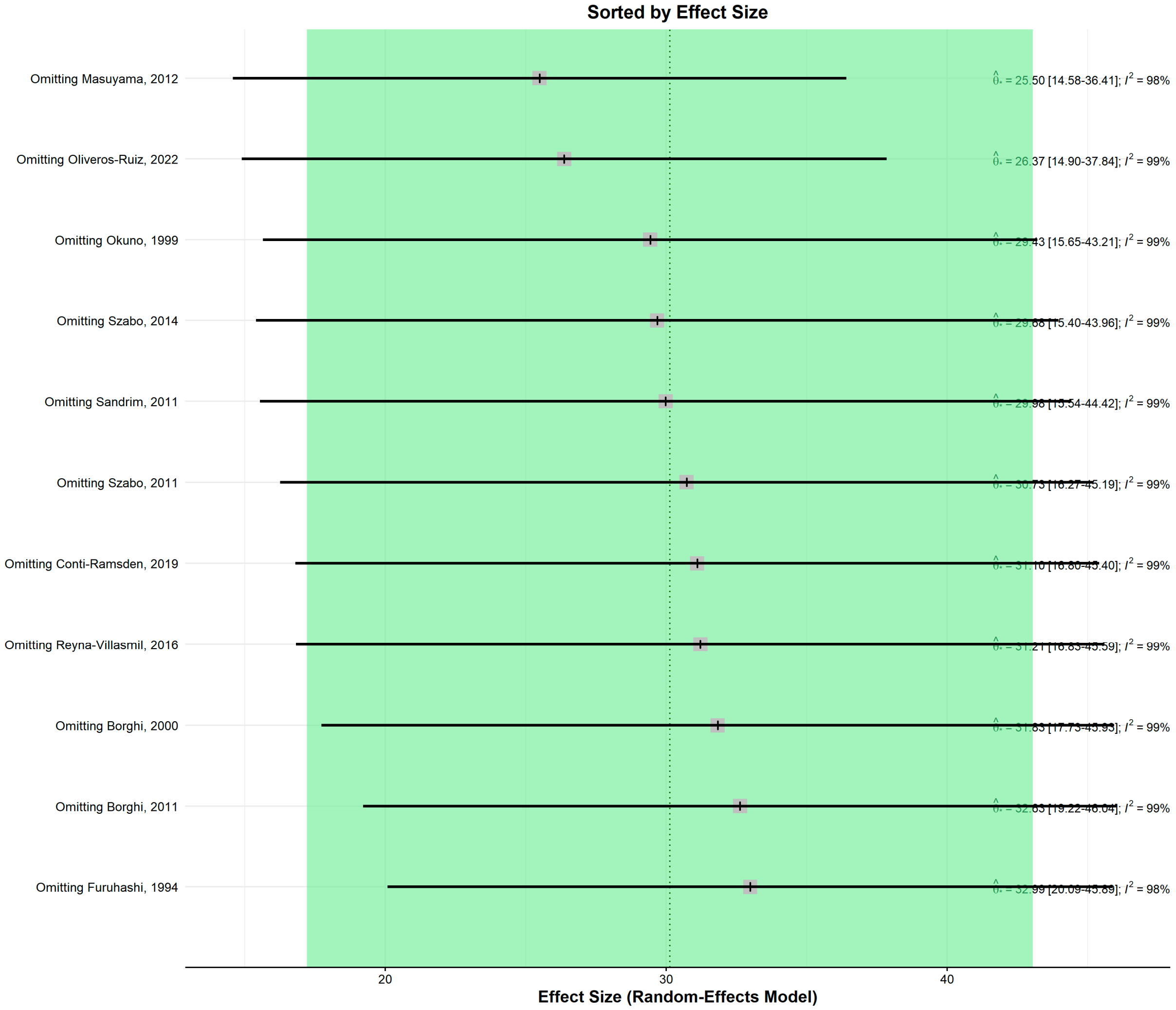

Supplementary Figure S3. Sensitivity analysis for BNP comparison between preeclampsia vs. healthy.

BNP, brain natriuretic peptide; Θ, mean difference [95% confidence interval]; I^2, inconsistancy index.

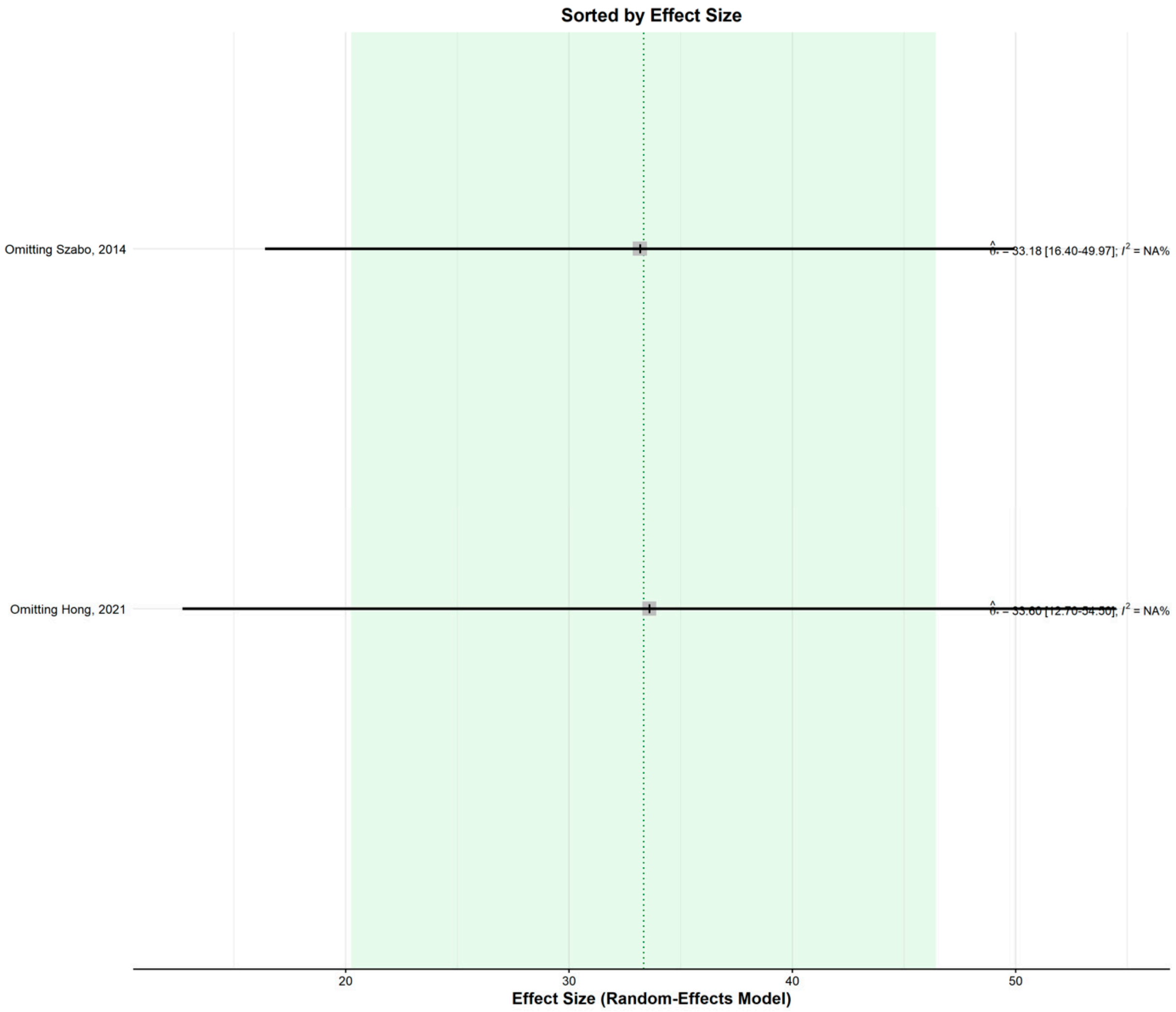

Supplementary Figure S4. Sensitivity analysis for BNP comparison between early onset preeclampsia (EOP) and late onset preeclampsia (LOP).

BNP, brain natriuretic peptide; Θ, mean difference [95% confidence interval]; I^2, inconsistancy index.

Supplementary Table S3. Newcastle Ottawa Scale quality assessment of the selected articles.

Author and year of publication	Cases DA	Cases R	Controls S	Controls D	Comparability	EA	Cases and controls A ϯ
Álvarez-Fernández, 2016	*	-	*	*	-	*	*
Bakacak, 2016	*	-	*	*	-	*	*
Barneo-Caragol, 2018	*	-	*	*	-	*	*
Borghi, 2011	*	*	*	*	matched age, gestational age	*	*
Borghi, 2000	*	-	*	*	-	*	*
Conti-Ramsden, 2019	^	^	*	^	^	*	*
Furuhashi, 1994	-	-	*	-	-	*	*
García Iglesias, 2022	*	*	*	*	-	*	*
Giannubilo, 2017	*	*	*	*	matched age, gestational age	*	*
Hamad, 2009	*	-	*	*	matched age, gestational age, no smoking	*	*
Hong, 2021	*	-	*	*	-	*	*
Jacobsen, 2022	*	-	*	*	-	*	*
Junus, 2014	*	-	*	*	-	*	*
Kale, 2005	*	-	*	*	-	*	*
Katja, 2013	^	^	*	^	^	*	*
Kumari, 2017	*	-	*	*	-	*	*
Lafuente-Ganuza, 2021	*	-	*	*	-	*	*
Masuyama, 2012	*	-	*	*	matched age, gestational age, parity, body mass index	*	*
Moghbeli, 2010	*	-	*	*	matched race	*	*
Okuno, 1999	*	-	*	*	matched age, gestational age, parity	*	*
Oliveros-Ruiz, 2022	-	*	*	-	-	*	*
Pankiewicz, 2020	*	-	*	*	-	*	*
Reyna-Villasmil, 2016	*	-	*	*	-	*	*
Sadlecki, 2016	*	-	*	*	-	*	*
Salimi, 2018	*	^	^	*	^	*	*
Sandrim, 2011	*	-	*	*	-	*	*
Seong, 2011	*	-	*	*	-	*	*
Szabó, 2014	*	*	*	*	matched age, body mass index, smoking	*	*
Szabó, 2011	*	-	*	*	-	*	*
Yu, 2018	*	-	*	*	-	*	*
Zhang, 2021	*	-	*	*	-	*	*

DA - definition adequacy; R – representativeness; D – definition; EA - exposure ascertainment; A – ascertainment; - according to design or analysis; ϯ - same method; * - fulfilled criteria; ^ -not reported.

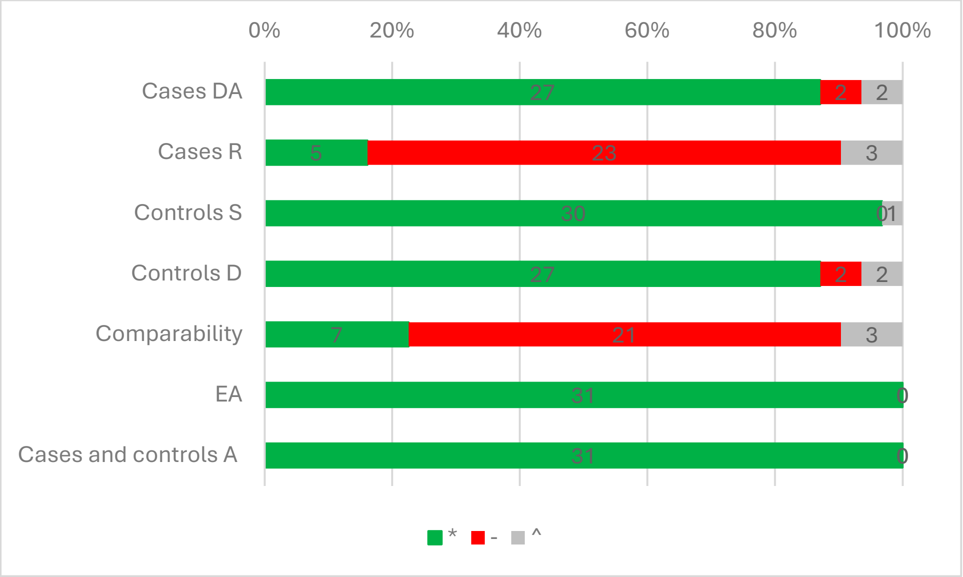

Supplementary Figure S5. Newcastle Ottawa Scale quality assessment of the selected articles.

DA - definition adequacy; R - representativeness; D - definition; EA - exposure ascertainment; A - ascertainment.

Publication bias

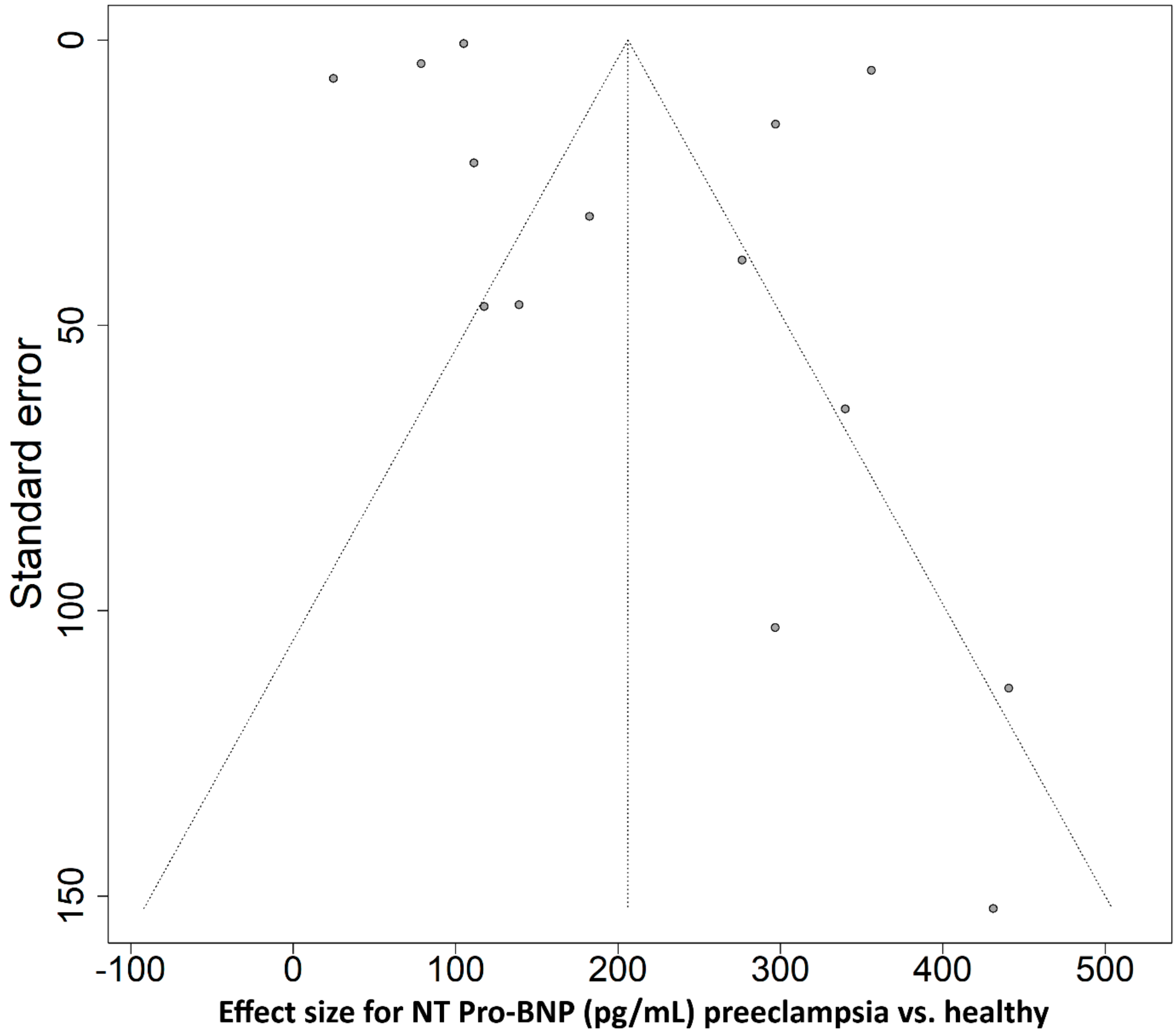

Supplementary Figure S6. Funnel plot for NT Pro-BNP comparison between preeclampsia vs. healthy.

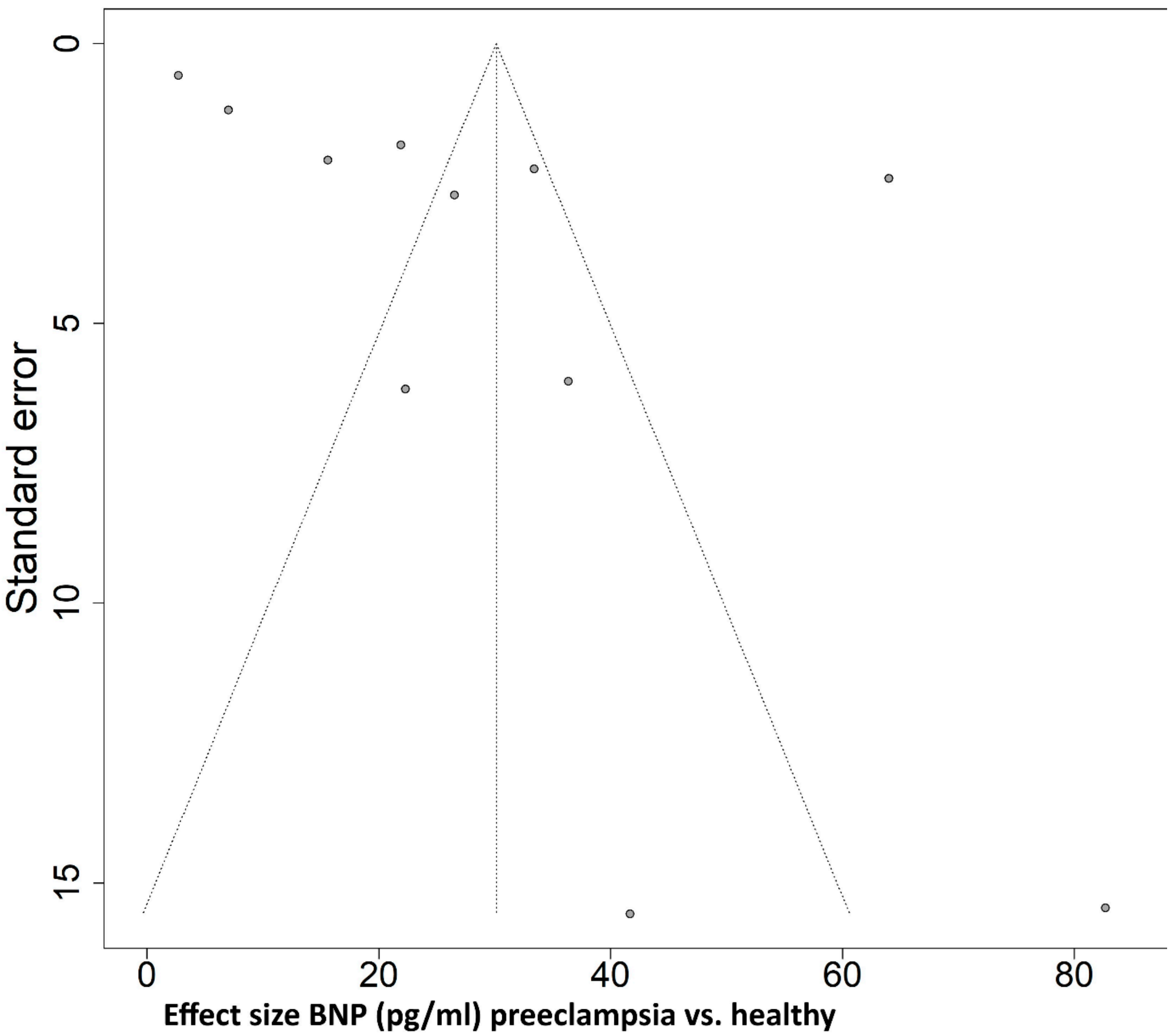

Supplementary Figure S7. Funnel plot for BNP comparison between preeclampsia vs. healthy.

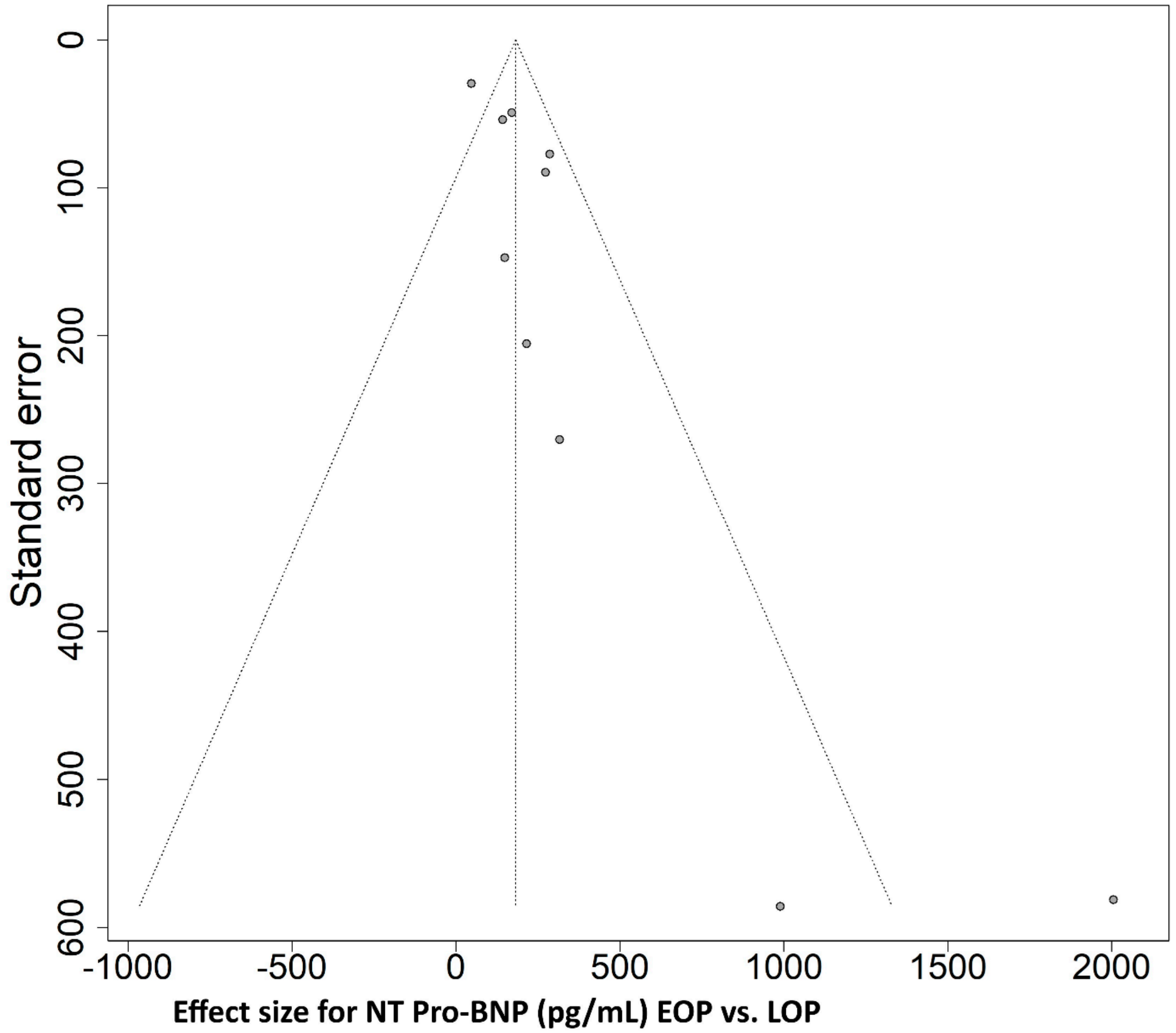

Supplementary Figure S8. Funnel plot for NT Pro-BNP comparison between early onset preeclampsia (EOP) and late onset preeclampsia (LOP).

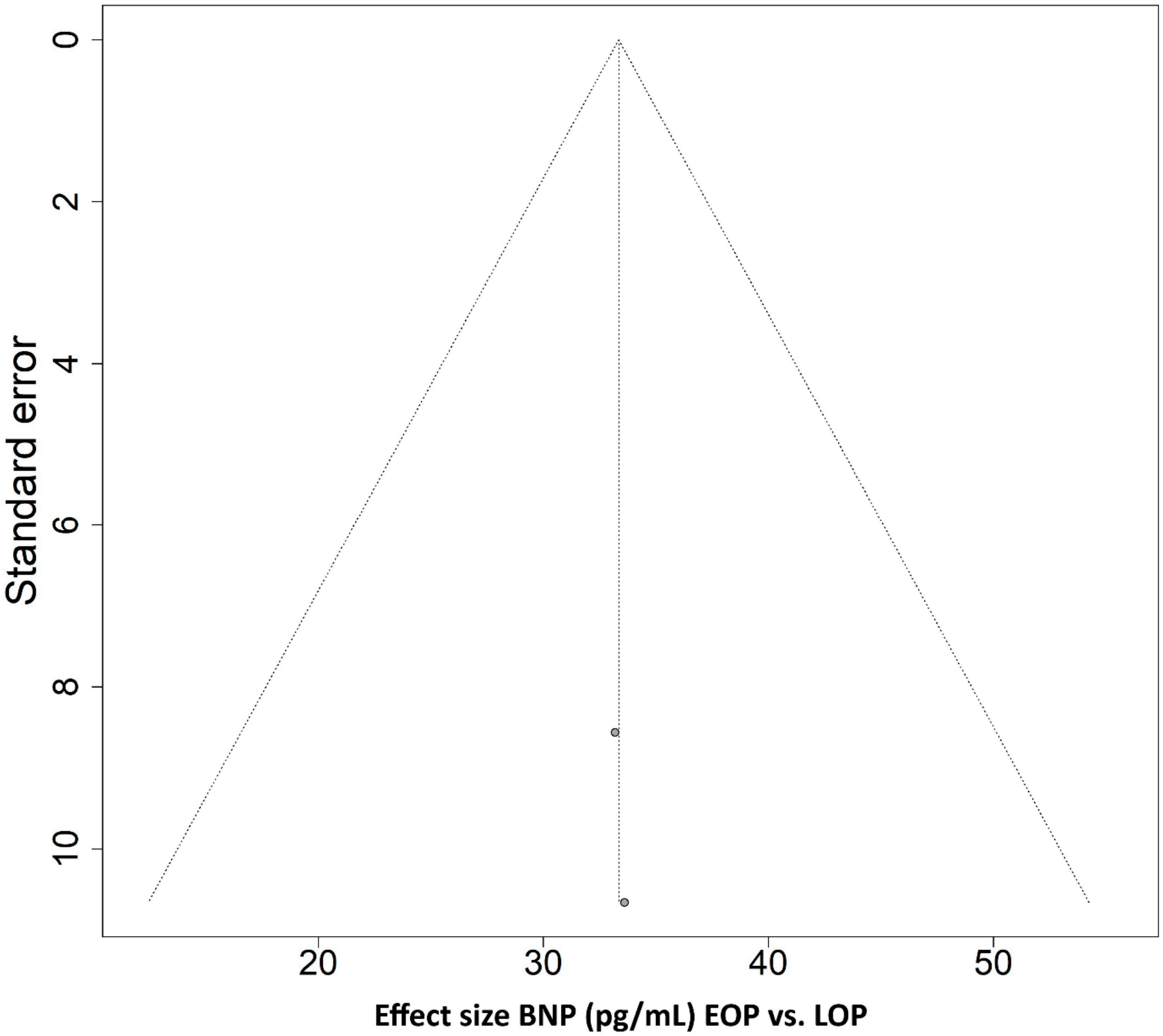

Supplementary Figure S9. Funnel plot for BNP comparison between early onset preeclampsia (EOP) and late onset preeclampsia (LOP).
